# Host specificity and species diversity of the Ostertagiinae Lopez-Neyra, 1947 in ruminants: a European perspective

**DOI:** 10.1186/s13071-018-2958-6

**Published:** 2018-06-28

**Authors:** Anna Wyrobisz-Papiewska, Jerzy Kowal, Paweł Nosal, Gabriela Chovancová, Steffen Rehbein

**Affiliations:** 1 Department of Environmental Zoology, Institute of Animal Sciences, University of Agriculture in Krakow, Mickiewicza av. 24/28, 30-059, Krakow, Poland; 2 Research Station and Museum of the Tatra National Park, 059 60 Tatranská Lomnica, Slovakia; 3 Merial GmbH, Kathrinenhof Research Center, Walchenseestr. 8-12, 83101 Rohrdorf, Germany

**Keywords:** Ostertagiinae, *Ostertagia leptospicularis*, Bovidae, Cervidae, Host specificity, Correspondence analysis

## Abstract

**Background:**

Nematodes of the subfamily Ostertagiinae appear to be rather specific to a species or family of hosts, but some are observed in a wide variety of hosts. The nematode *Ostertagia leptospicularis* draws special attention due to its presence or absence among the same host species in different European countries. Therefore, this paper focuses mainly on the host specificity among nematodes of the subfamily Ostertagiinae. The second aim of this study is to assess the possibility of treating *O. leptospicularis* as an *Ostertagia* species complex.

**Methods:**

Data were gathered from *post*-*mortem* examinations of domestic and wild ruminants (*n* = 157), as well as bibliographical references (*n* = 96), which were pooled and discussed. The research area was limited to European countries, hence the studied ostertagiine species are limited to native ones; likewise, the host species. Special emphasis was placed on the mean abundance values that allowed a typical host or hosts for each nematode species to be specified. Correspondence analysis was performed to confirm the stated host specificity.

**Results:**

The analysis revealed that nematodes of this subfamily tend to use ruminants from a particular subfamily as their principal host. The results indicate that *Ostertagia leptospicularis*, similar to *Teladorsagia circumcincta*, may represent a potential species complex. This nematode, as the sole member of the subfamily Ostertagiinae, occurs in almost all representatives of the Bovidae subfamily, as well as in the Cervidae.

**Conclusions:**

Despite the stated narrow host specificity, the results obtained may suggest that *O. leptospicularis* is not strongly connected to any host or is comparably associated with a very wide and diverse group of hosts (Cervidae, Bovidae). The *Ostertagia* complex may have particular cryptic species or strains typical for any individual host or group of hosts. Such a conclusion requires further investigations on a wider scale.

## Background

Parasites are able to colonize one or more host species, and therefore can be classified as specialists, specific for a species or a family of hosts, or generalists, capable of infecting a wide variety of hosts [1]. Generally, their unambiguous distinction is supported by morphological features; however, in some cases their species specialization started to be questioned. Nematodes of the order Strongylida, particularly those of the subfamily Ostertagiinae, are the most common parasites of ruminants [2]. They constitute a very diverse group of parasites with a similar morphology that include particular features, which may reflect genuine species differences or just morphological variations within the same species [3].

The ostertagiine taxonomy is complicated, mainly for the species of *Teladorsagia*. Results of advanced morphological analyses confirmed by molecular techniques revealed a significant intraspecific diversity in the populations of these nematodes. Until recently, *Teladorsagia circumcincta* (Stadleman, 1894) was treated as a single species that occurs in various hosts (both domestic and wild ruminants). However, some studies on the genus *Teladorsagia* have demonstrated the existence of two distinct strains within its domestic hosts. The first one was found in sheep and goats, while the other occurred only in goats. In the case of wild ruminants (i.e. muskoxen, barrenground caribou and peary caribou), the nematode regarded previously to be *T. circumcincta* was discovered to be a distinct species, named *Teladorsagia boreoarcticus* Hoberg, Monsen, Kutz & Blouin, 1999 to emphasize its origin from a geographically isolated region [4]. Consequently, it is currently believed that the aforementioned member of the subfamily represents the *Teladorsagia* species complex, with particular cryptic species (or strains) [5]. Even less is known about the occurrence of cryptic species in other frequently occurring representatives of the Ostertagiinae, especially in *Marshallagia marshalli* Ransom, 1907, due to its presence in several host species [6]. Similar doubts may also be raised in relation to the genus *Ostertagia*; however, such data are not yet available [5].

Generalist species seem to be able to easily colonize different host populations and pose a serious health risk due to their higher pathogenic effect [7]. Nematodes of Ostertagiinae appear to be rather specific for a species or family of hosts; however, some of them are observed in a wide variety of hosts. *Ostertagia leptospicularis* Assadov, 1953 especially seems to be a generalist, but such a statement requires broader analysis. From an epizootiological point of view, it is desirable to know which parasite species are more prone to interspecific transmission (i.e. host switching) [8]. Such knowledge would allow an evaluation to be made of possible health risks in situations when different host populations (domestic and wild) may share the same pasture or in the case of animal migration. Collecting valid and comparable data on the biodiversity of ruminant nematodes will be possible when we take into account the aforementioned host specificity and proper species identification [5].

The aim of the present study is to evaluate host specificity among Ostertagiinae, and assess the possibility of treating *O. leptospicularis* as an *Ostertagia* species complex.

## Methods

### Analysis strategy

Several parasitological approaches were combined for this investigation. The first step was the *post-mortem* (*pm*) examination of domestic and wild ruminants from southern Poland, Slovakia and Germany to characterize the parasitic fauna. Only results on the subfamily Ostertagiinae were taken into account. The second step focused on collecting similar data from other European countries and pooling them with those obtained from the *pm* examinations. The third step was performing the correspondence analysis.

### *Post-mortem* examination and parasite identification

The *pm* examinations were carried out on 157 animals (Table 1), according to parasitological procedures [9]. The study provided a unique data on parasitic fauna of Tatra chamois and fallow deer, and allowed the gathering of outcomes that not only correspond with previously published studies, but also significantly expand the data suitable for further analyses. All of the recovered nematodes were identified on the basis of morphological features [10–13]. Males of the Ostertagiinae were determined to species, females only to subfamily (the concept of polymorphism was included). The taxonomy of the polymorphic males among the genera and species of abomasal nematodes within the Ostertagiinae follows Dróżdż [13]. The proper intensity of the infection was calculated by multiplying the data on males by the observed sex ratio (males: females), hence the results provided in Table 1 and Table 3 apply to both sexes.

**Table 1:** Number of nematodes of the Ostertagiinae subfamily from the post-mortem examinations of ruminant species

Family	Subfamily	Species	Origin	No. of animals examined			No. of nematodes recovered
Bovidae	Bovinae	Cattle (*Bos taurus*)	Poland, Germany^a^	11		59	955
	Caprinae	Domestic sheep (*Ovis aries*)	Poland	32	48		57
		Tatra chamois (*Rupicapra rupicapra tatrica*)	Slovakia	16^b^			882
Cervidae	Capreolinae	Roe deer (*Capreolus capreolus*)	Poland, Slovakia	84		98	4065
	Cervinae	Fallow deer (*Dama dama*)	Poland	6	14		142
		Red deer (*Cervus elaphus*)	Poland, Slovakia	8			25
Total						157	6126

^a^Only abomasum

^b^The digestive tract of one Tatra chamois had no abomasum

### Data collection

Additional data were collected from 96 bibliographical references (along with some unpublished data from Germany), in which at least one species of the subfamily Ostertagiinae derived from domestic or wild ruminants during the *pm* examination was mentioned (coproscopical analyses, experimental infections, and the *pm* examination of wild ruminants kept in outdoor enclosures were excluded). The number of animals included in the further analysis is shown in Table 2. The research area included only European countries, hence the studied ostertagiine species are limited to the native ones, i.e. *Ostertagia leptospicularis* / *O. kolchida* Popova, 1937; *O. ostertagi* (Stiles, 1892) / *O. lyrata* Sjoberg, 1926; *O. antipini* Matschulsku, 1950 / *O. lyrateformis* (Dróżdż, 1965); *O. drozdzi* Jancev, 1977 / *O. ryjikovi* (Jancev, 1977); *O. gruehneri* Skrjabin, 1920 */ O. arctica* Mitzkewitsch, 1929; *Teladorsagia circumcincta* / *T. trifurcata* (Ransom, 1907); *Spiculopteragia boehmi* (Gebauer, 1932) / *S. mathevosiani* Ruchliadev, 1948; *S. asymmetrica* (Ware, 1925) / *S. quadrispiculata* (Jansen, 1958); *S. houdemeri* (Schwartz, 1926) */ S. andreevae* (Dróżdż, 1965); *Mazamastrongylus dagestanica* (Altaev, 1953); *M. marshalli / M. occidentalis* Ransom, 1907. The majority of the references were gathered using the Web of Science Core Collection citation database.

**Table 2:** Number of animals included in the analysis of the bibliographical references

Family	Subfamily	Species	No. of animals		
Bovidae	Bovinae	Cattle (*Bos taurus*)	1281	1398	2814
	Caprinae	European bison (*Bison bonasus*)	117		
		Domestic sheep (*Ovis aries*)	455	1416	
		Mouflon (*Ovis musimon*)	191		
		Domestic goat (*Capra hircus*)	160		
		Alpine ibex (*Capra ibex*)	163		
		Pyrenean ibex (*Capra pyrenaica*)	79		
		Chamois (*Rupicapra rupicapra*)	368		
Cervidae	Capreolinae	Roe deer (*Capreolus capreolus*)	1315	1436	3600
		Moose (*Alces alces*)	98		
		Reindeer (*Rangifer tarandus*)	23		
	Cervinae	Red deer (*Cervus elaphus*)	1,439	2164	
		Sika deer (*Cervus nippon*)	471		
		Fallow deer (*Dama dama*)	254		

### Data analysis

The data obtained from the *pm* examinations and the literature were analyzed by pooling the prevalence (P) and all values relating to the intensity of infection (i.e. mean, maximum and minimum). Special emphasis was placed on the mean abundance (MA), i.e. the total number of individuals of a particular parasite species in a sample of a particular host species, regardless of whether or not the host was infected. MA is equivalent to the mean intensity multiplied by P, and therefore is very valuable if biodiversity is considered [14].

In case of ostertagiine species, the identification of males (m) is more reliable; thus, most of the analyzed data applied only to them. However, the total number of females (f) was calculated if the mean intensity of infection and sex ratio were determined. Consequently, it was possible to calculate the actual intensity of females and males in a species for some bibliographical references. As the majority of the papers provided information about the mean intensity of infection, the formula ∑f + ∑m / examined animals or ∑m / examined animals, where ∑f and ∑m are the summed intensity of each sex in a host sample was applied, and the MA was calculated and used in further analyses that allowed the principal host/hosts for each nematode species to be specified. If a major and minor form of one species was included separately, only the major one was counted with the total intensity calculated for both.

### Statistical analysis

The correspondence analysis was performed using Statgraphics Centurion XVI software to confirm host specificity. The MA was used as the measure characterizing the association between the rows and columns in the contingency table.

## Results

### *Post-mortem* examination and parasite identification

The results obtained from the *pm* examinations of 157 animals are shown in Table 3. Overall, 6,126 specimens (Table 1) belonging to eight species of the Ostertagiinae were found: *O. leptospicularis*, *O. ostertagi*, *O. antipini*, *O. drozdzi*, *T. circumcincta*, *S. asymmetrica*, *S. boehmi* and *M. marshalli*. Four of the species (approximately 4050 specimens) were derived from roe deer. The majority of nematode species were found in more than one host species; however, *S. antipini* and *M. marshalli* only occurred in roe deer and Tatra chamois, respectively. *Spiculopteragia boehmi* was the most common parasite species in the Cervidae family, whereas *T. circumcincta* was the most common in the Bovidae. The prevalence varied from 1.3% (*S. asymmetrica* infecting roe deer) to 100% (*S. boehmi,* roe deer, as well as *O. leptospicularis* and *O. ostertagi*, cattle). The highest intensity of infection (reaching 829 specimens) with *O. ostertagi* was in cattle, and the lowest (just 3 specimens) with *M. marshalli* in Tatra chamois. Interestingly, *O. leptospicularis* was the only species that was found in both the representatives of the Bovidae (i.e. cattle and sheep) and Cervidae (i.e. roe deer).

**Table 3:** Nematode species of the Ostertagiinae subfamily from the examined animals during post mortem examinations

Host species/ Nematode species	Bovidae			Cervidae			Origin
	Bovinae	Caprinae		Capreolinae	Cervinae		
	Cattle (*Bos taurus*)	Domestic sheep (*Ovis aries*)	Tatra chamois (*Rupicapra rupicapra tatrica*)	Roe deer (*Capreolus capreolus*)	Red deer^a^ (*Cervus elaphus*)	Fallow deer^a^ (*Dama dama*)	
*Ostertagia leptospicularis*		P = 1/32; I = 5		P = 48/80; I = 46 (1–469)			Poland
				P = 4/4; I = 79 (17–203)			Slovakia
	P = 1/1; I = 102						Germany
*O. ostertagi*	P = 3/10; I = 380 (80–740)						Poland
	P = 1/1; I = 829						Germany
*O. antipini*				P = 5/80; I = 11 (3–23)			Poland
*O. drozdzi*						P = 1/6; I = 147	Poland
*Teladorsagia circumcincta*		P = 5/32; I = 62 (3–43)					Poland
			P = 14/15; I = 62 (2–191)				Slovakia
*Spiculopteragia asymmetrica*				P = 1/80; I = 4		P = 5/6; I = 25 (2–104)	Poland
*S. boehmi*				P = 32/80; I = 40 (1–463)	P = 2/6; I = 13 (2–23)	P = 2/6; I = 19 (5–33)	Poland
				P = 4/4; I = 38 (10–94)			Slovakia
*Marshallagia marshalli*			P = 2/15; I = 3 (3–4)				Slovakia

^a^Not all of the dissected animals were infected. One fallow deer from Poland, as well as two red deer from Slovakia were free of ostertagiine species

*Abbreviations*: *P* prevalence of infection given as no. of infected/examined animals, *I* mean (minimum, maximum) intensity of infection

### Data collection and analysis

The comparison of bibliographical data collected showed the differences in the occurrence of ostertagiine species among European ruminant host species (Table 4). According to the references available, the hosts of the family Bovidae were studied by means of *pm* examinations in 17 countries (those of the subfamily Caprinae in 15, Bovinae in 8), whereas Cervidae in 16 countries (Capreolinae in all 16, Cervinae in 9). The most abundant data were available for roe deer, while those for European bison, Alpine ibex, Pyrenean ibex and reindeer were limited due to their narrow range of occurrence.

**Table 4:** Occurrence of Ostertagiinae among European ruminant host species, based on bibliographical references

Host			Country	Nematode species										
Family	Subfamily	Species		*Ol*	*Oo*	*Oa*	*Od*	*Og*	*Tc*	*Sa*	*Sb*	*Sh*	*Md*	*Mm*
Bovidae	Bovinae	Cattle (*Bos taurus*)	Poland		*+*									
			Germany	+	*+*				+		+			
			Austria	+	*+*				+		+			
			Czechoslovakia		+									
			Belgium	+	+									
			Netherlands	+	+				+					
			United Kingdom		+									
			Hungary		+									
		European bison (*Bison bonasus*)	Poland	+	+	+				+	+			
	Caprinae	Domestic sheep (*Ovis aries*)	Poland	+	+				+					
			Germany	+					+					
			Czech Republic						+					
			Czechoslovakia		+				+		+			
			Italy	+	+				+					+
			Norway						+					
			Finland					+	+					
			United Kingdom						+					
		Mouflon (*Ovis musimon*)	Germany	+	+				+	+	+			
			Czechoslovakia	+	+				+		+			
			Netherlands	+					+		+			
			Italy		+				+					+
			Spain						+					
		Domestic goat (*Capra hircus*)	Poland		+				+					
			Germany						+		+			
			Lithuania						+					
			France		+				+					
			Italy	+	+				+					+
			Norway						+					
		Alpine ibex (*Capra ibex*)	Germany	+					+					+
			Austria	+	+				+		+			+
			Switzerland	+	+				+					+
		Pyrenean ibex (*Capra pyrenaica*)	Spain		+				+					+
		Chamois (*Rupicapra rupicapra*)	Germany	+	+				+		+			+
			Austria	+	+				+		+			+
			Czechoslovakia		+				+		+			
			Slovakia						+					+
			Italy	+					+		+			
Cervidae	Capreolinae	Roe deer (*Capreolus capreolus*)	Poland	+	+	+			+	+	+		+	
			Germany	+	+				+	+	+	+		
			Austria	+	+						+	+		
			Czechoslovakia	+	+				+		+			
			Slovakia	+							+			
			Ukraine											+
			Netherlands	+	+				+		+			
			France	+	+						+			
			Italy	+	+				+		+			+
			Spain						+					
			Sweden	+	+				+					
			United Kingdom	+	+						+			
			Croatia		+				+					
		Moose (*Alces alces*)	Poland	+		+					+		+	
			Norway	+		+			+				+	
			Finland	+		+							+	
			Sweden	+		+			+		+		+	
			Russia (European part)			+				+			+	
		Reindeer (*Rangifer tarandus*)	Finland					+	+					
	Cervinae	Red deer (*Cervus elaphus*)	Poland	+	+		+		+	+	+		+	
			Germany	+			+		+	+	+	+		
			Austria	+							+	+		
			Czechoslovakia		+				+		+			
			Italy	+					+		+			+
			Spain	+	+		+		+	+				
			Norway	+							+			
			United Kingdom	+						+	+			
			Croatia						+					
		Sika deer (*Cervus nippon*)	Poland	+						+	+			
			Germany	+						+		+		
			Austria	+							+	+		
			Czechoslovakia						+	+	+			
		Fallow deer (*Dama dama*)	Poland	+			+			+	+			
			Germany	+	+					+	+			
			Austria	+			+			+	+			
			Czechoslovakia	+	+				+		+			
			Italy				+			+				
			Spain	+			+			+				
			United Kingdom	+						+	+			

*Abbreviations*: *Md* *Mazamastrongylus dagestanica*, *Mm* *Marshallagia marshalli*, *Oa* *O. antipini*, *Od* *O. drozdzi*, *Og* *O. gruehneri*, *Ol* *Ostertagia leptospicularis*, *Oo* *O. ostertagi*, *Sa* *Spiculopteragia asymmetrica*, *Sb* *S. boehmi*, *Sh* *S. houdemeri*, *Tc* *Teladorsagia circumcincta*

The parasitic fauna of European cervids consists of eleven species of the Ostertagiinae, and therefore can be considered as more diverse than that of the bovids (eight species); *O. drozdzi*, *S. houdemeri* and *M. dagestanica* are observed only in cervids. Within the countries studied, the most commonly occurring species were *O. leptospicularis* and *T. circumcincta*, found in 12 and 13 host species, respectively. Conversely, *O. drozdzi* and *O. gruehneri* were found only in two.

In many countries, *O. leptospicularis* was found in almost all representatives of the family Cervidae, with most reports concerning roe deer. Wild and domestic ruminant hosts from Poland and Germany had very similar parasitic faunas. Nevertheless, they varied in some findings, including those referring to *O. leptospicularis*. The majority of the differences presented concerned the composition of ruminant fauna in both countries (i.e. free-living European bison and moose in Poland, as well as mouflon and chamois in Germany). Despite the broad examination of cattle in both countries, *O. leptospicularis* was noted only in Germany (similarly to Austria, Belgium and the Netherlands). Lack of this nematode in Polish cattle, or in cattle from other European countries, infers that more attention should be dedicated to this species.

### Statistical analysis

**Fig. 1:**
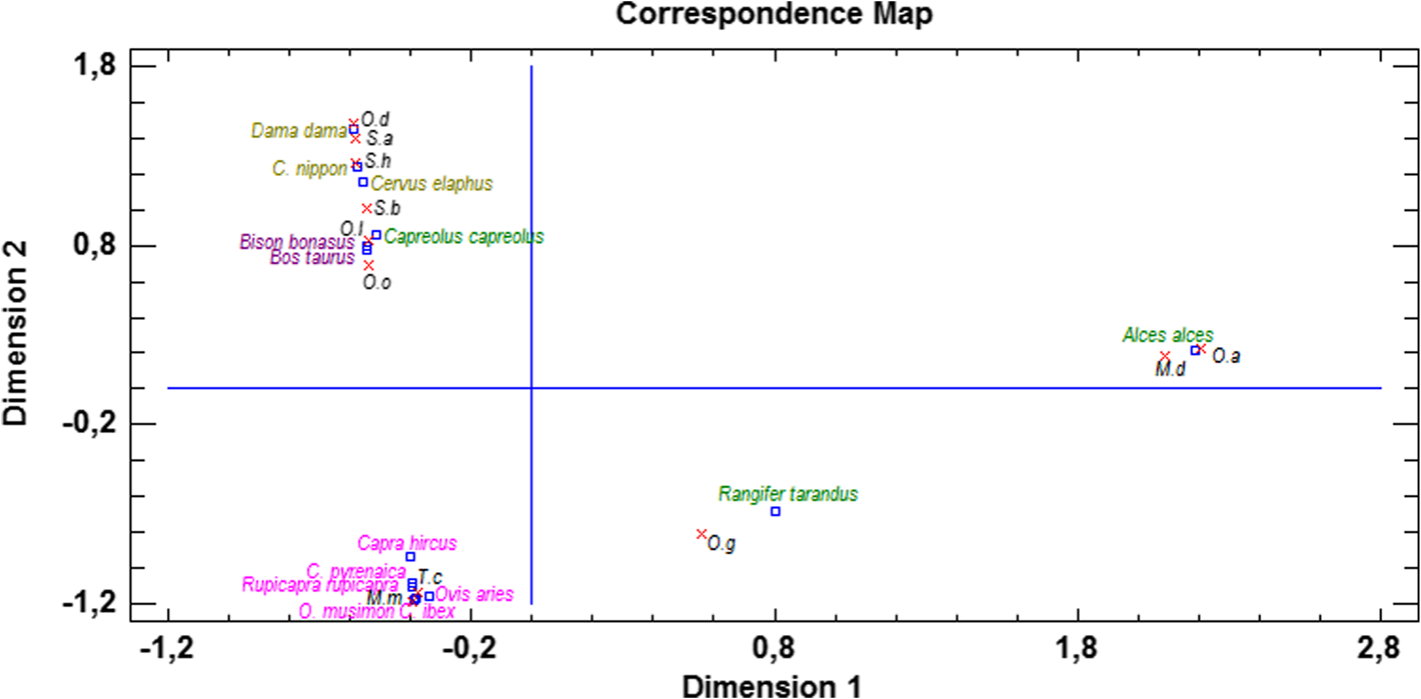
Parasite-host relationships among European ruminant host species on the basis of mean abundance (symmetric map of the correspondence analysis). Red crosses, nematode species; blue squares, host species. Subfamilies of ruminants are highlighted by colors: Bovinae, purple; Caprinae, pink; Capreolinae, green; Cervinae, olive

**Fig. 2:**
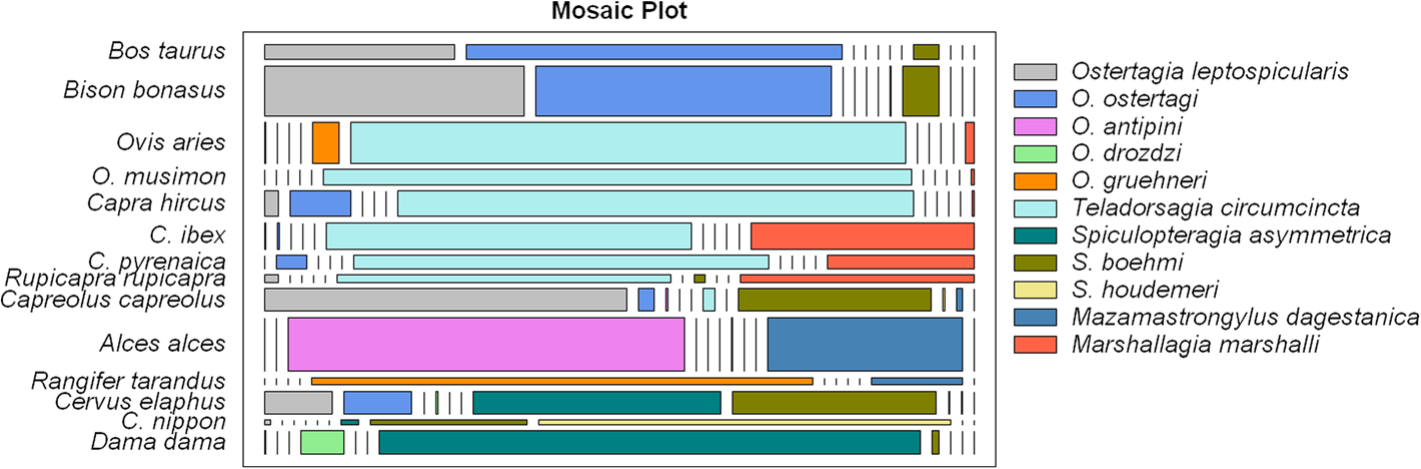
Host specificity of Ostertagiinae nematodes among European ruminant host species based on the prevalence. Length of the rectangles corresponds to the strength of the connection; width corresponds to the number of observations

**Fig. 3:**
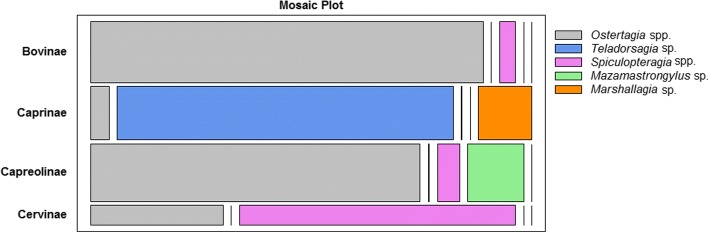
Relation between the particular ostertagiine genus and its hosts (at the subfamily level). Length of the rectangles corresponds to the strength of connection; width corresponds to the number of observations

Both the correspondence map (Fig. 1), as well as the mosaic plots (Figs. 2, 3) explain 59.4529% of the variability amongst the rows and columns.

The correspondence analysis divided the Ostertagiinae and their hosts into clearly distinct groups (Fig. 1). Based on the results, it can be stated that *M. marshalli* and *T. circumcincta* are most strongly associated with small ruminants (i.e. species of the subfamily Caprinae). This close connection is also observed between *O. ostertagi* and other bovids (i.e. those of the subfamily Bovinae). However, cattle seem to be a more typical host (the distance between the cross and square is shorter for *O. ostertagi* and cattle than for *O. ostertagi* and the European bison). Nematodes of the genus *Spiculopteragia*, as well as *O. drozdzi*, show the strongest link to Cervinae representatives, while *O. gruehneri*, *O. antipini* and *M. dagestanica* to those of the subfamily Capreolinae (i.e. reindeer and moose, respectively).

The strongest connections between a parasite and individual host was between *O. antipini* and moose, *S. houdemeri* and sika deer, as well as *O. drozdzi*, *S. asymmetrica* and fallow deer (red crosses are almost inscribed in the blue squares, Fig. 1). These hosts can be treated as principal hosts (rather than auxiliary hosts).

The distance from *O. leptospicularis* to roe deer and European bison on the correspondence map seems to be almost equal (Fig. 1). Furthermore, *O. leptospicularis* as the sole member of the subfamily Ostertagiinae occurs in the majority of the Bovidae, as well as Cervidae (Fig. 2).

It should also be pointed out that the ostertagiine of a particular genus are most strongly connected to hosts from a particular subfamily (Table 5). Figure 3 shows the strong relationship between *Teladorsagia* sp. / *Marshallagia* sp. and Caprinae, *Spiculopteragia* spp. and Cervinae, as well as *Mazamastronyglus* sp. and Capreolinae. A strong connection between *Ostertagia* spp. and Bovinae can be observed, but this genus is also significantly related to Capreolinae. Furthermore, cervids are the hosts of nematodes that are more specialized than those parasitizing bovids (Table 5).

**Table 5:** Host specificity of the nematodes of the Ostertagiinae subfamily among European ruminant host species

Typical host			Nematode species
Family	Subfamily	Species	
Bovidae	Bovinae	Cattle (*Bos taurus*)	*Ostertagia ostertagi*; *O. leptospicularis*
		European bison (*Bison bonasus*)	*Ostertagia ostertagi*; *O. leptospicularis*
	Caprinae	Domestic sheep (*Ovis aries*)	*Teladorsagia circumcincta* (species complex)
		Mouflon (*Ovis musimon*)	*Teladorsagia circumcincta*
		Domestic goat (*Capra hircus*)	*Teladorsagia circumcincta* (species complex)
		Other *Capra* species (*Capra* sp.)	*Teladorsagia circumcincta*; *Marshallagia marshalli*
		Chamois (*Rupicapra rupicapra*)	*Teladorsagia circumcincta*; *Marshallagia marshalli*
Cervidae	Capreolinae	Roe deer (*Capreolus capreolus*)	*Ostertagia leptospicularis*
		Moose *(Alces alces)*	*Ostertagia antipini*; *Mazamastrongylus dagestanica*
		Reindeer (*Rangifer tarandus*)	*Ostertagia gruehneri*
	Cervinae	Red deer (*Cervus elaphus*)	*Spiculopteragia boehmi*
		Fallow deer (*Dama dama*)	*Spiculopteragia asymmetrica*; *Ostertagia drozdzi*

The analysis conducted allowed the principal host/hosts to be defined for all species of the Ostertagiinae (Table 5).

## Discussion

Some ruminant species have already been treated as the principal host of Ostertagiinae nematodes, but only on the basis of observations of *pm* results (especially P). Such data do not provide a clear overview of host specificity. According to these outcomes, members of the subfamilies Bovidae and Cervidae have different helminth fauna, characterized by specific dominant species, which depends on several factors (e.g. climate, host co-occurrence and introduction of alien host species).

Among bovids, the most frequently found nematodes are *T. circumcincta*, *O. ostertagi* and *M. marshalli*. The first two are the most important gastrointestinal parasites of domestic ruminants [15–17]. *Teladorsagia circumcincta* is the dominant species in all bovids, while *M. marshalli* seems to be almost entirely restricted to hosts that are well adapted to a cold climate (i.e. representatives of chamois and ibex) [7]. In the case of wild members of the family Bovidae, the European bison is thought to be a host of the same Ostertagiinae species as is found in domestic ruminants. It is hard to define whether the European bison is a primary or secondary host for these nematodes or is a principal host for any of them. The exchange of helminths between European bison and representatives of the family Cervidae is also worth mentioning. Due to their co-occurrence, European bison adopted *O. leptospicularis* and *S. boehmi* from roe and red deer and in this way enriched the parasitic fauna of the abomasal nematodes with those typical for representatives of the family Cervidae [18, 19].

Cross-transmission is highly visible among cervids, and therefore these hosts are successfully used for studies on formatting the parasitic fauna of ruminants. All representatives of the family Cervidae are the principal hosts of particular ostertagiine species. The origin of such a relationship is dated to over ten million years ago, when the cervids of the subfamily Paleomericinae divided into two lineages, and since then, nematodes and their hosts evolved in parallel. A high degree of co-evolution is also seen among bovids [19, 20]. The presence of a principal host may affect parasites spreading to sympatric cervid or bovid hosts. Species of the genus *Spiculopteragia* are generally restricted to cervids, although records of infected bovids in Europe have been documented [21]. Fallow deer is the principal host of *S. asymmterica*; however, it can be found in sympatric species, such as red deer, roe deer or even European bison if they co-occur with the principal host [22]. The same applies to *O. antipini*, *M. dagestanica* and the largest species of Cervidae, moose, which has currently arisen as its principal host [23]. These nematodes can parasitize other wild ruminants only if the territory is inhabited by the principal host [18, 24, 25]. Furthermore, alien host species (e.g. sika deer) may also affect the diversity of the parasitic fauna of native hosts, and lead to the emergence of new diseases. Interestingly, the decomposition of parasitic fauna of alien host species has also been observed, e.g. the sika deer introduced to Poland have adopted some nematode species from local cervids, but have simultaneously lost several of their typical parasites [24].

Representatives of the family Cervidae (especially red deer and moose) migrate seasonally, which encourage the possibility of parasite transmission not only to other wild ruminants, but also to livestock [19, 26]. This poses a health threat to domestic ruminants in areas where they graze in pastures used by wild hosts, since every untypical infection may lead to high pathogenicity. It is also worth pointing out that such pathogenicity can significantly increase if co-infections with nematodes of the subfamily Ostertagiinae are considered [27]. On the other hand, wild ruminants can be exposed to clinical diseases after becoming infected with nematodes that are rather related to bovids [28]. Moreover, their parasitic fauna can be enriched with drug-resistant strains, contributing to the spread of such strains [29].

*Ostertagia leptospicularis* circulates among cervids, cattle and sheep. This species is not very host-specific; nevertheless, it seems to have mostly adopted roe deer [7, 16]. Based on the results obtained, *O. leptospicularis* was noted as having become an outstanding and unique species. The equal distance from *O. leptospicularis* to roe deer and European bison on the correspondence map may suggest that this nematode is not strongly connected to any of the hosts, or that it may be comparably associated with very a wide and diverse groups of hosts.

## Conclusions

The analyses conducted confirmed that nematodes of the Ostertagiinae appear to be specific for a species or family of hosts (i.e. ostertagiines of a particular genus are most strongly connected to hosts from a particular subfamily). For the majority of them, a principal host can be indicated, except for *O. leptospicularis*. *Ostertagia leptospicularis* seems to have a principal host among members of both subfamilies the Bovidae and Cervidae. If the confirmed host specificity can be considered an indication of the species diversity among the Ostertagiinae in ruminants, then *O. leptospicularis* could be treated as a candidate for an *Ostertagia* species complex. Further investigations (morphological and molecular) should firmly establish if, as with *T. circumcincta,* this nematode represents another species complex with particular cryptic species/strains typical for any individual host/group of hosts, or, as the sole member of the subfamily Ostertagiinae, is a generalist, capable of infecting a wide variety of hosts.

## Abbreviations

MA: Mean abundance
P: Prevalence
*pm*: *post-mortem*
